# Medication-Related Osteonecrosis Successfully Treated With Hyperbaric Oxygen Therapy and Conservative Treatment: A Case Report

**DOI:** 10.7759/cureus.79213

**Published:** 2025-02-18

**Authors:** Tougo Tanabe, Taku Kimura, Ken-ichiro Sakata, Noritaka Ohga, Aya Yanagawa-Matsuda, Yoshinori Kobori

**Affiliations:** 1 Department of Oral Diagnosis and Medicine, Hokkaido University, Sapporo, JPN; 2 Department of Vascular Biology and Molecular Pathology, Faculty of Dental Medicine and Graduate School of Dental Medicine, Hokkaido University, Sapporo, JPN; 3 Department of Oral Surgery, Azabu Triology Hospital, Sapporo, JPN

**Keywords:** conservative treatment, denosumab, hyperbaric oxygen therapy (hbo therapy), medical collaboration, medication-related osteonecrosis of the jaws (mronj)

## Abstract

Medication-related osteonecrosis of the jaws (MRONJ) is a major side effect of the antiresorptive drugs used in treating patients with osteoporosis and cancer. MRONJ appears as prolonged inflammation affecting the maxilla or mandible, with necrotic bone exposure and intraoral and extraoral fistulas. According to previous studies, surgery for this condition is highly beneficial. However, extensive surgical treatment generally overburdens patients with MRONJ, who often already suffer from other diseases such as cancer; therefore, further consideration must be given to their overall condition. Herein, we present the case of an elderly patient with extensive MRONJ who was successfully treated with hyperbaric oxygen therapy and conservative treatment.

## Introduction

Medication-related osteonecrosis of the jaw (MRONJ) is a persistent inflammatory condition primarily associated with androgen receptor antagonists (ARAs) prescribed for cancer and osteoporosis treatment [1]. The global prevalence of MRONJ is on the rise, with occurrence rates varying between 0.02% and 18%, influenced by the patient's medical background, such as the cumulative dosage of ARAs, especially bisphosphonates (BPs) and antibody against receptor activator of nuclear factor kappa-B ligand (RANKL) and the length of therapy [1-3]. As stated in a position document by the American Association of Oral and Maxillofacial Surgeons, MRONJ is classified into four distinct stages (Stages 0-3) according to clinical and imaging evaluations [1]. Treatment strategies for MRONJ can either be conservative (using antimicrobial treatment and oral irrigation) or surgical (such as marginal and segmental jaw resections) [2]. In patients with severe MRONJ (Stage 2 or 3), surgical treatment is more beneficial than conservative treatment modalities such as sequestrectomy and saucerization [3,4,5,6]. However, extensive surgery often places a heavy burden on patients with MRONJ, who often have poor surgical tolerance due to their multiple comorbidities. Moreover, even if extensive surgery is performed on such patients, there is a risk of postoperative infection, which may lead to a poor prognosis. Therefore, in many cases, surgery performed under general anesthesia is either not applicable or undesirable. Therefore, oral surgeons sometimes have to opt for other treatment modalities despite the good treatment outcomes obtainable with surgery. Hyperbaric oxygen (HBO) therapy is increasingly utilized in the management of a range of conditions, such as osteoradionecrosis of the jaw (ORN), persistent inflammatory disorders in the oral and maxillofacial area, chronic diabetic foot ulcers, and serious systemic inflammation [7-10]. It has been reported that HBO therapy for patients with MRONJ offers a modest benefit alongside surgical or conservative treatment compared with either treatment alone [8,9,11]. However, to our knowledge, there is a lack of studies showing the effectiveness of HBO therapy for these patients, and HBO has remained an adjuvant therapy. Recently, we have been able to assess the impact of HBO therapy on patients with advanced MRONJ quantitatively through nuclear medicine imaging techniques, such as fluorodeoxyglucose-positron emission tomography (FDG-PET) and bone single-photon emission computed tomography, successfully developing a treatment plan involving HBO for patients with advanced MRONJ [12]. These therapeutic approaches involve HBO therapy as well as conservative methods such as antibiotic therapy, oral irrigation, and minimally invasive surgery, and we have previously reported cases of elderly patients with MRONJ in which extensive surgery was avoided using this treatment strategy [13]. This report presents the case of an older patient with advanced MRONJ who was effectively managed by conservative treatment such as HBO therapy and saucerization.

## Case presentation

**Figure 1:**
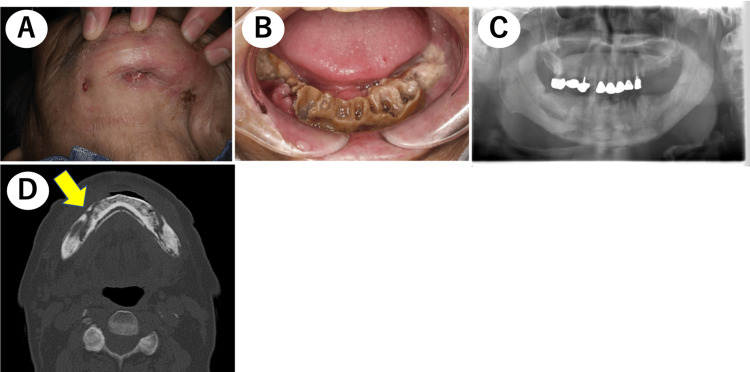
Clinical and radiographic findings before the treatment. (A) The extraoral examination revealed a fistula on the skin of the right mandibular area. (B) The intraoral examination revealed exposed bone extending from the lower right molar area to the lower left molar area. (C) The panoramic X-ray revealed signs of sequestration, consistent with the exposed bone area. (D) The axial MDCT image revealed widespread sequestration and abnormal sclerotic changes in the mandibular alveolar bone (the yellow arrow points to indications of sequestrum in the mandibular bones). MDCT, multidetector computed tomography

An 81-year-old male with a history of bone metastasis of prostate cancer, who had been treated with denosumab for 34 months, was sent to our department due to extensive mandibular bone exposure. Denosumab had been administered as 120 mg subcutaneously once every four weeks during this period, according to the prescribed dosage. Two months before visiting our department, denosumab was changed to another prostate cancer drug (antiandrogen) after consultation with the hospital’s dentist and urologist, prioritizing treatment for osteonecrosis of the jaw. His extraoral examination revealed an extraoral fistula in the skin over his right mandible (Figure 1A), while his intraoral examination revealed exposed bone extending from the lower right molar area to the lower left molar area (Figure 1B). His panoramic X-ray revealed signs of sequestration, consistent with the exposed bone area (Figure 1C). Multidetector computed tomography (MDCT) revealed widespread sequestration and abnormal sclerotic changes in the mandibular alveolar bone (Figure 1D). According to these findings, the patient was diagnosed with Stage 3 MRONJ in both mandibular bones.

**Figure 2:**
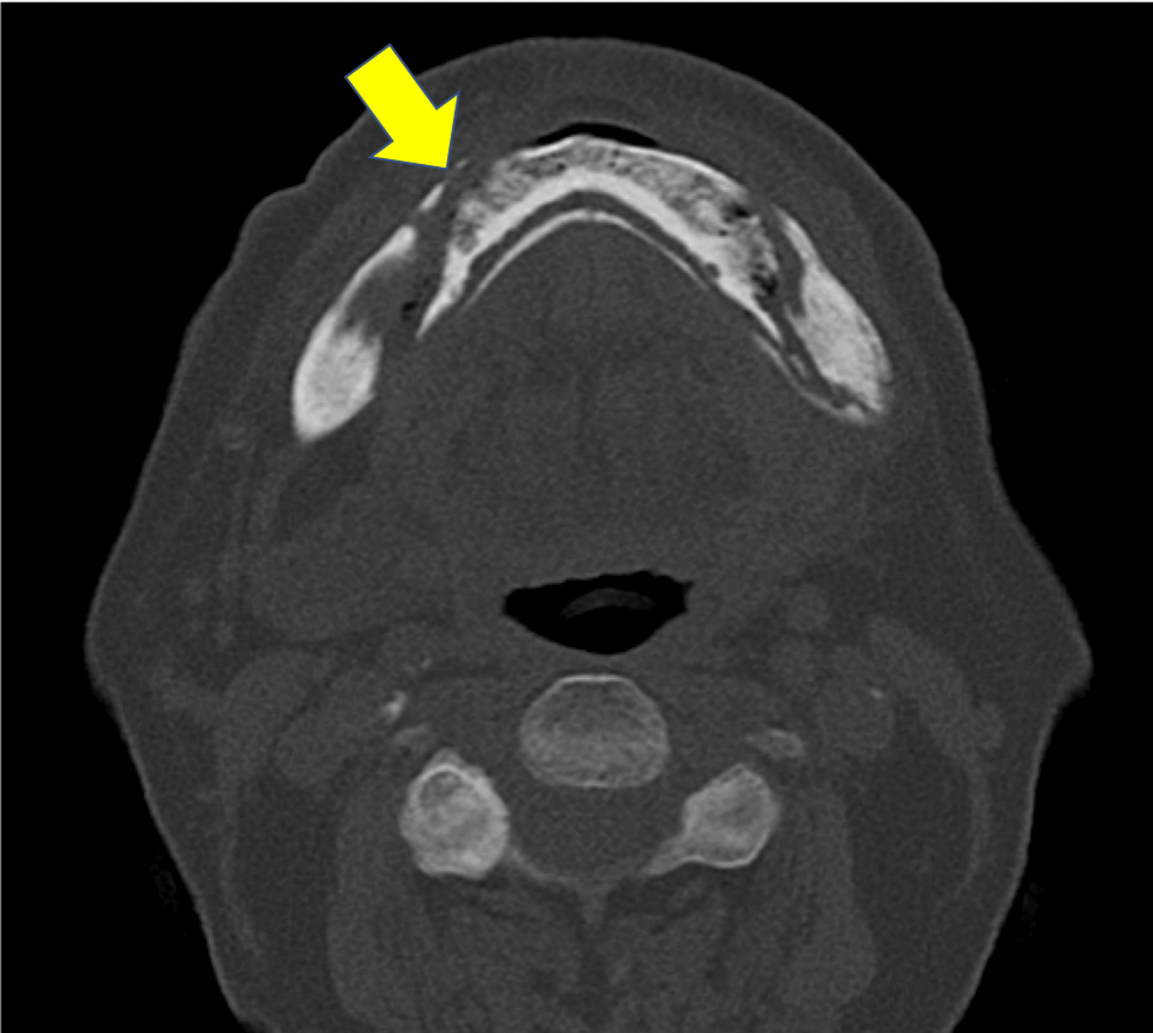
Radiographic finding after HBO treatment. The axial MDCT image showed increased separation of the sequestrum compared to the initial one (the yellow arrow indicates signs of sequestrum separation). MDCT, multidetector computed tomography; HBO, hyperbaric oxygen

Surgical treatment modalities such as reconstructive surgery were considered appropriate; however, considering the patient’s poor tolerance to surgery and the wishes of his family, conservative treatment was preferred. The patient received antimicrobial treatment with oral amoxicillin (AMPC) alone at a dose of 750 mg/day for 60 days and underwent regular oral irrigation. During this period, he also received HBO therapy 20 times on an outpatient basis. No side effects from long-term use of antibacterial drugs were observed. In our department, the HBO therapy protocol consisted of compression to 2.4 absolute atmospheres (0.146 MPa gauge pressure) at a steady rate for 15 minutes, followed by pressure maintenance for 60 minutes, and gradual decompression to normal pressure over 20 minutes using a hyperbaric oxygen chamber (KHO-301B, Kawasaki Engineering, Kobe, Japan) [13]. After HBO treatment, the control MDCT showed increased separation of the sequestrum compared with the initial one (Figure 2).

**Figure 3:**
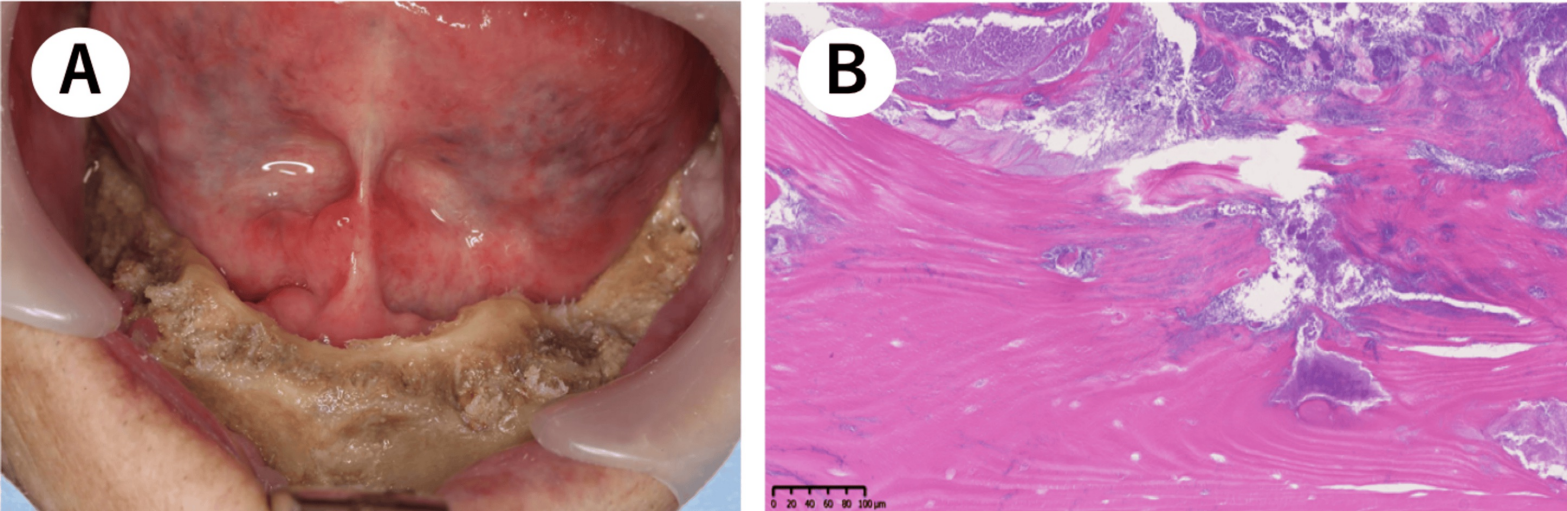
Saucerization. (A) Intraoral finding after saucerization. (B) Pathological examination revealed a sequestrum with granulation tissue and bacterial colonies.

Thereafter, to improve the cleaning of the exposed bone areas, he underwent saucerization under local anesthesia (Figure 3A). The subsequent histopathological assessment detected a sequestrum accompanied by bacterial colonies and granulation tissue, which served as a basis for making MRONJ the final diagnosis (Figure 3B).

**Figure 4:**
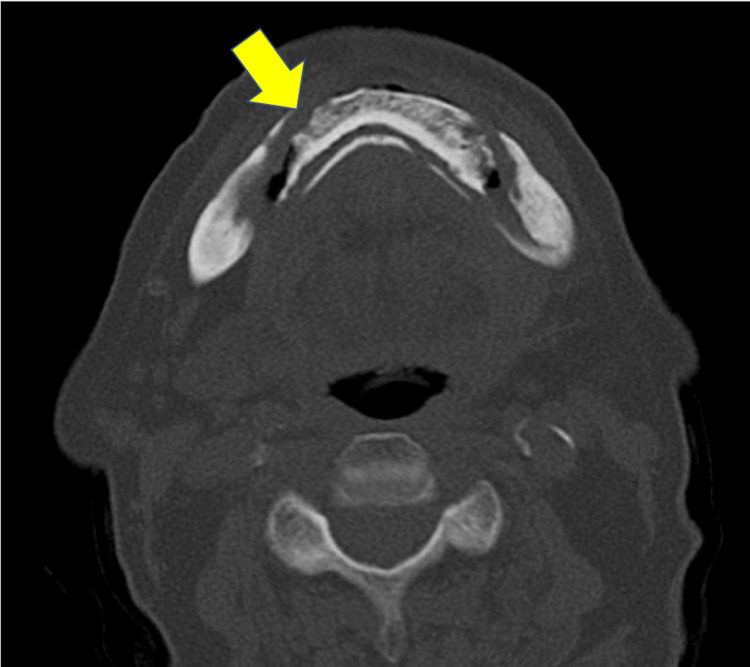
Radiographic finding nine months after the initial visit. Axial MDCT image showed a trend toward further separation of the sequestrum compared with the second one (the yellow arrow indicates the signs of separation of the sequestrum). MDCT, multidetector computed tomography

The patient received oral AMPC at a dose of 750 mg/day for 30 days as the sole antimicrobial drug after saucerization. He also underwent oral irrigation regularly at the hospital’s dentistry unit every two weeks and denture adjustments at his family dental office every one or two months. The third MDCT, which was performed nine months after the initial visit, showed a trend toward further separation of the sequestrum compared with the second one (Figure 4).

**Figure 5:**
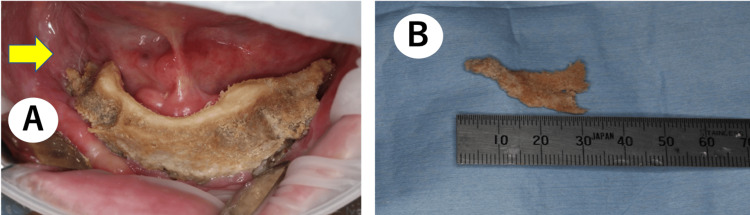
Sequestrectomy. (A) Intraoral finding after sequestrectomy: the bone sequestration area indicated by the yellow arrow was removed. (B) Extracted sequestrum.

Thereafter, because the exposed sequestrum of the right mandible had increased mobility, the patient underwent sequestrectomy under local anesthesia (Figures 5A, 5B).

**Figure 6:**
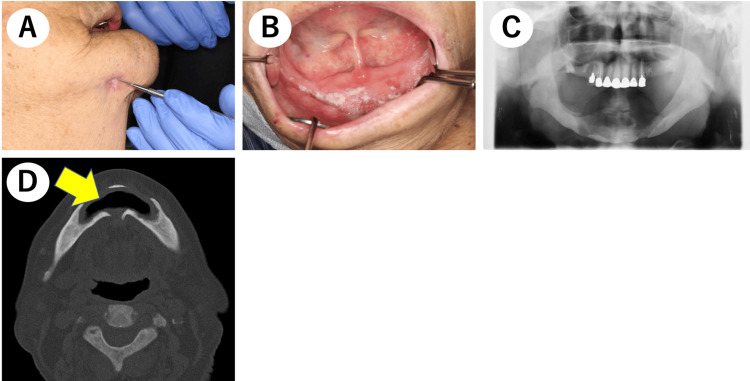
Clinical and radiographic findings after the remaining sequestrum was completely shed naturally. (A) The extraoral examination revealed a fistula but no signs of pus drainage. (B) The intraoral examination revealed no signs of bone exposure or cavities. (C) Panoramic X-ray examinations revealed smooth margins of the alveolar bones in the mandibular areas. (D) The axial MDCT image showed complete shedding of the sequestrum (the yellow arrow). MDCT, multidetector computed tomography

The mucosa under the area where the sequestrum was removed showed no signs of bone exposure or cavities. After that, the patient continued oral irrigation at the hospital’s dentistry unit and had adjustments made to the denture he had been using since the beginning at his family dental office. Consequently, two years and four months after denosumab was discontinued, all of the remaining sequestrum was naturally shed. There was no acute phase of infection during the course of treatment. The mucosa under the area where the sequestrum had fallen revealed no signs of bone exposure or cavity (Figure 6A). While an extraoral fistula persisted, there were no signs of pus drainage from the extraoral fistula and gingiva in the oral region (Figure 6B) . Panoramic X-ray and MDCT examinations revealed the smooth margins of the alveolar bones in the mandibular areas (Figures 6C, 6D).

## Discussion

**Figure 7:**
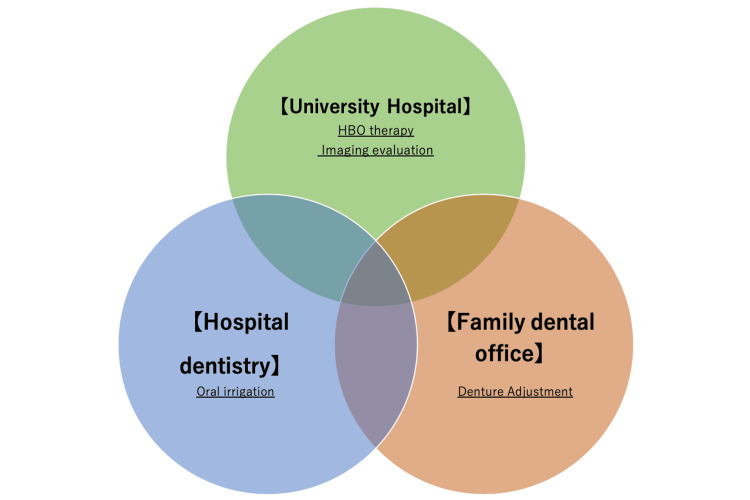
Medical collaboration among three dental clinics: the university hospital, hospital dentistry, and family dental office. Image credit: Tougo Tanabe. HBO, hyperbaric oxygen

Here, we report a case of an elderly patient with advanced MRONJ who was successfully treated with a combination of conservative therapy and HBO treatment. To date, HBO therapy has been proven effective in the prevention and treatment of ORN [11]. In managing patients undergoing HBO therapy, it is necessary to consider the side effects commonly associated with the treatment. Essentially, in HBO therapy, patients are placed in a chamber where the pressure is 1.5 to 3.0 times greater than that of normal air. Therefore, patients with claustrophobia and pulmonary conditions are not candidates for HBO therapy [14]. According to prior research, the adverse effects of HBO therapy include barotrauma affecting the middle ear and paranasal sinuses, ear discomfort, as well as ocular complications such as myopic shifts [14,15]. Therefore, clinicians should consider these potential side effects when putting patients on HBO therapy. In this case, the patient did not exhibit any side effects throughout the course of treatment. The mechanisms of HBO therapy could provide clinical advantages, such as (1) enhanced wound healing by promoting angiogenesis, (2) antibacterial effects through the generation of reactive oxygen species via oxygenation of the affected tissue, and (3) improved blood circulation and reduced hypoxia by decreasing gas accumulation caused by edema under high pressure [12,16]. In other words, HBO therapy promotes angiogenesis, allowing oxygen and antibiotics to reach the MRONJ lesions and promote healing. Prior research has also indicated that patients with MRONJ who underwent HBO therapy exhibit a bone healing process that differs from normal bone remodeling, which is known as mini modeling [12]. This unique process, which occurs by regenerating new bone on top of the existing bone, is shown as a smooth border between the existing and regenerated bone. In this case, in addition to the impact of HBO therapy on the patient, discontinuing denosumab may have contributed to the cure of MRONJ. Denosumab was administered to inhibit osteoclast-mediated bone resorption in bone metastases, and the administration of this drug may have contributed to the pathogenesis of MRONJ. Drugs that can cause MRONJ (like denosumab) include the so-called BPs, such as zoledronic acid; however, they have different mechanisms of action and pharmacokinetics. Denosumab specifically inhibits RANKL, suppressing osteoclast formation and activation; however, this inhibition is reversible. BPs, conversely, inhibit bone resorption primarily by directly suppressing farnesyl diphosphate synthase activity in the mevalonate pathway. As a result, the cytoskeleton of the osteoclasts is destroyed, osteoclast apoptosis is enhanced, and osteoclast function is reduced. From a pharmacokinetic perspective, BPs are rapidly removed from plasma and are distributed intensively in the bone [17]. They are irreversibly deposited on the mineralizing bone surface at bone mineralization sites and osteoclast resorption sites for a long period even after being discontinued. The half-life of BPs in the bone matrix exceeds 10 years after deposition. Therefore, there remains some doubt regarding the efficacy of discontinuing BPs for the management of MRONJ, as it may have limited effectiveness. However, denosumab, which does not accumulate in the bone, is gradually cleared from the bloodstream after discontinuation. Thus, because the half-life of denosumab is significantly shorter than that of BPs, the discontinuation of the former may result in the separation of a sequestrum, which may be effective in improving MRONJ [18]. Ohga et al. [19] and Malan et al. [20] reported cases of MRONJ caused by denosumab in which there was spontaneous healing after the completion of denosumab treatment. As in this case, if the primary tumor is properly controlled and denosumab can be discontinued, its discontinuation and the adoption of conservative treatment methods, including HBO, may allow for the successful cure of MRONJ. However, since factors such as the resumption of denosumab may lead to the recurrence of MRONJ, lifelong follow-up in collaboration with the patient's primary care physician is essential. In this case, medical collaboration among the three dental clinics was required to achieve the patient’s conservative treatment (Figure 7). 

**Figure 8:**
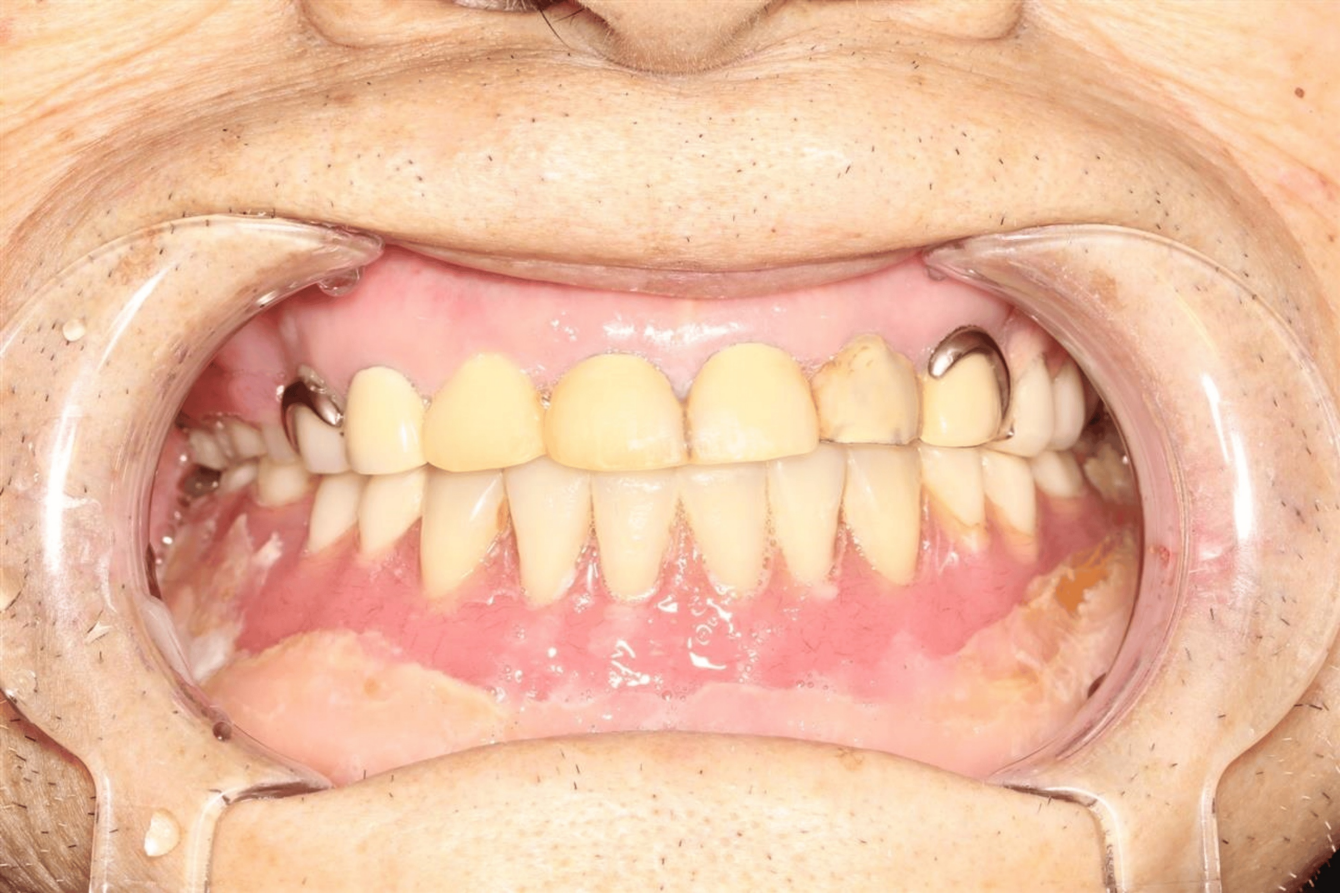
Dentures that patients had continued to use during treatment. With proper medical collaboration among the three dental clinics, the patient was able to continue using his dentures.

At his family dental office, his dentures were adjusted every one to two months, and he underwent oral irrigation regularly at the hospital’s dentistry unit every two weeks. At the university hospital (our hospital), he received HBO therapy and underwent imaging evaluations every six months. The patient was cured successfully while fulfilling the wishes of his family to avoid surgery. Furthermore, he continued having his denture adjusted at his family dental office and was, therefore, able to continue using it until the sequestrum was completely shed (Figure 8). As in this case, collaboration among multiple medical institutions can greatly contribute to maintaining the quality of life of older patients.

## Conclusions

MRONJ is a refractory condition that occasionally necessitates extensive surgical intervention. Herein, we reported the case of an older patient with advanced MRONJ who was adequately managed by conservative treatment with HBO therapy, avoiding extensive surgery. This case also shows that discontinuing denosumab may contribute to improving MRONJ. Furthermore, through our case, continued medical collaboration enabled the continued use of dentures and contributed to the maintenance of the patient’s quality of life.
